# Mortality and thermal environment (UTCI) in Poland—long-term, multi-city study

**DOI:** 10.1007/s00484-020-01995-w

**Published:** 2020-09-02

**Authors:** Magdalena Kuchcik

**Affiliations:** Climate Impacts Laboratory, Institute of Geography and Spatial Organization Polish Academy of Sciences, Twarda 51/55, 00-818 Warszawa, Poland

**Keywords:** Thermal environment, UTCI, Mortality, Poland, 40 years study

## Abstract

The aim of the study was to establish to what extent extreme thermal conditions have changed and how they affected mortality, and what conditions favor lower mortality rates or conversely, higher mortality rates. Heat/cold exposure was measured with the Universal Thermal Climate Index (UTCI). Daily mortality and meteorological data for 8 large Polish cities (Białystok, Gdańsk, Kraków, Lublin, Łódź, Poznań, Warszawa, and Wrocław) in the period 1975–2014 were analyzed. Generalized additive models were used to investigate the relationship between UTCI and mortality, and TBATS models were used for the evaluation of time series UTCI data. Most of the cities experienced a clear and statistically significant at *p* ≤ 0.05 decrease in cold stress days of 0.8–3.3 days/year and an increase in the frequency of thermal heat stress days of 0.3–0.6 days/year until 1992–1994. There was a clear difference as regards the dependence of mortality on UTCI between cities located in the “cooler” eastern part of Poland and the “warmer” central and western parts. “Cool” cities were characterized by a clear thermal optimum, approx. in the range of 5–30 °C UTCI, changing slightly depending on cause of death, age, or sex. For UTCI over 32 °C, in most of the cities except Gdańsk and Lublin, the relative risk of death (RR) rose by 10 to 20%; for UTCI over 38 °C, RR rose to 25–30% in central Poland. An increase in mortality on cold stress days was noted mainly in the “cool” cities: RR of total mortality increased even by 9–19% under extreme cold stress.

## Introduction

The atmospheric environment affects human beings constantly in a limited but clear way. Climate features are mortality risk factors alongside key issues such as genetics, lifestyle, and environmental factors (including sanitary conditions and air pollution) (EHR 2012). However, in many situations, extreme weather becomes the main risk factor, leading indirectly or even directly to death. As climate change has started manifesting itself, e.g., in the rise of air temperature and the frequency and intensity of heat periods (IPCC 2014, 2018), interest in analyses of mortality as dependent on thermal conditions has increased. This interest grew stronger after the record warm August of 2003: in this heat wave, approximately 45,000 additional deaths were recorded in Western and Southern Europe (e.g., Michelozzi et al. 2004; Garssen et al. 2005; Filleu et al. 2006; Robine et al. 2008).

Generally, the greatest impact on the human body by climate elements is caused by the thermal environment, which consists of solar radiation, temperature and humidity, and wind speed, while the exchange of heat between the body and the environment is the most important process that determines proper functioning of the body. Under extreme weather conditions, these factors become the main risk factor, leading indirectly and sometimes directly to death, which is widely described in many books and papers (Tromp 1980; Guyton and Hall 2006; Cheshire 2016).

A hot environment is a significant burden on the cardiovascular system. By provoking changes in blood flow and blood pressure, it may cause significant weakness of the body, headaches and dizziness, fever, disruption and loss of consciousness, and ultimately death. Heat lasting several days leads to a decrease in the amount of oxygen-carrying hemoglobin; as a result, the respiration rate increases, which is dangerous for those suffering from respiratory diseases (Tromp 1980; Kenney 1998; Noe et al. 2012).

In a cold environment, the first reaction of the thermoregulatory system is to constrict blood vessels, which results in a lower skin temperature. When this is not enough, the mechanism of shivering and non-shivering thermogenesis is activated. This generates even 4–5 times more heat than normally. However, it requires a greater blood supply to the skeletal muscles and causes loss of heat from inside the body. When the body loses more heat than it produces, tissues are damaged, muscles stiffen, and tidal volume decreases; loss of consciousness, ventricular fibrillation, and heart failure follow, leading to death (LeBlanc 1987; Kaciuba-Uscilko and Greenleaf 1989; Pozos and Danzl 2002).

In Poland, there is plentiful research on air temperature changes, describing the trend of rising air temperature in Warszawa by 0.7 °C/100 years over the years 1780–2012 (Lorenc 2000; KLIMADA 2014), or the decrease in the number of cold days (*T*_max_ ≤ − 10 °C) by 3.6 days/10 years in the period 1951–2010 and the clear increase in the frequency of days with *T*_max_ > 25 °C by 2.2 days/10 years, or nights with *T*_min_ > 18 °C by 2.4 days/10 years after 1990 (Twardosz and Kossowska-Cezak 2013). The frequency of variously defined hot and cold periods is also often examined (Kozłowska-Szczęsna et al. 2004; Wibig et al. 2009a, 2009b; Kuchcik et al. 2013; Krzyżewska 2015).

While meteorological data, especially from synoptic stations, are readily available in Poland, there is a difficulty to obtain long series of mortality data. Previous biometeorological studies covered 1–10-year-long periods of mortality, morbidity, or work/car accidents, usually in one city (Baranowska and Gabryl-Wojtach 1987; Śmietanka 1998; Kuchcik 2001, 2003; Krzyżewska et al. 2015; Rabczenko et al. 2016) or several cities (Kozłowska-Szczęsna et al. 2004; Błażejczyk and McGregor 2007; Rabczenko et al. 2009; Kuchcik 2017; Błażejczyk et al. 2018). This is the first such long analysis (covering 40 years) based on daily data concerning the thermal environment and mortality in Poland.

Several indicators of heat/cold exposure exist, as reviewed by Epstein and Moran (2006), Błażejczyk et al. (2012), and de Freitas and Grigorieva (2017). Among indexes, the Universal Thermal Climate Index (UTCI) finally developed 10 years ago (Bröede et al. 2012; Fiala et al. 2012; Jendritzky et al. 2012) has gained high interest and wide use in climate-mortality studies (Błażejczyk et al. 2014, 2015; Urban and Kyselý 2014; Kuchcik 2017; di Napoli et al. 2018).

In light of the above, the main hypothesis of this research was “extreme thermal conditions generate an increase in mortality, causing death directly or indirectly by exacerbating diseases; this increase varies between cities with different climates”. The main questions were as follows: to what extent did extreme thermal conditions affect mortality? What conditions are connected with lower mortality rates and what conditions, with an increase in mortality? To check these hypotheses, changes in the frequency of heat/cold stress categories (UTCI) were determined over the years 1975–2014, statistical trends for these changes and their statistical significance were calculated, the relative risk of death due to thermal environment as defined with UTCI was calculated, and thresholds accompanying rises in mortality were assessed.

## Material and methods

**Fig. 1:**
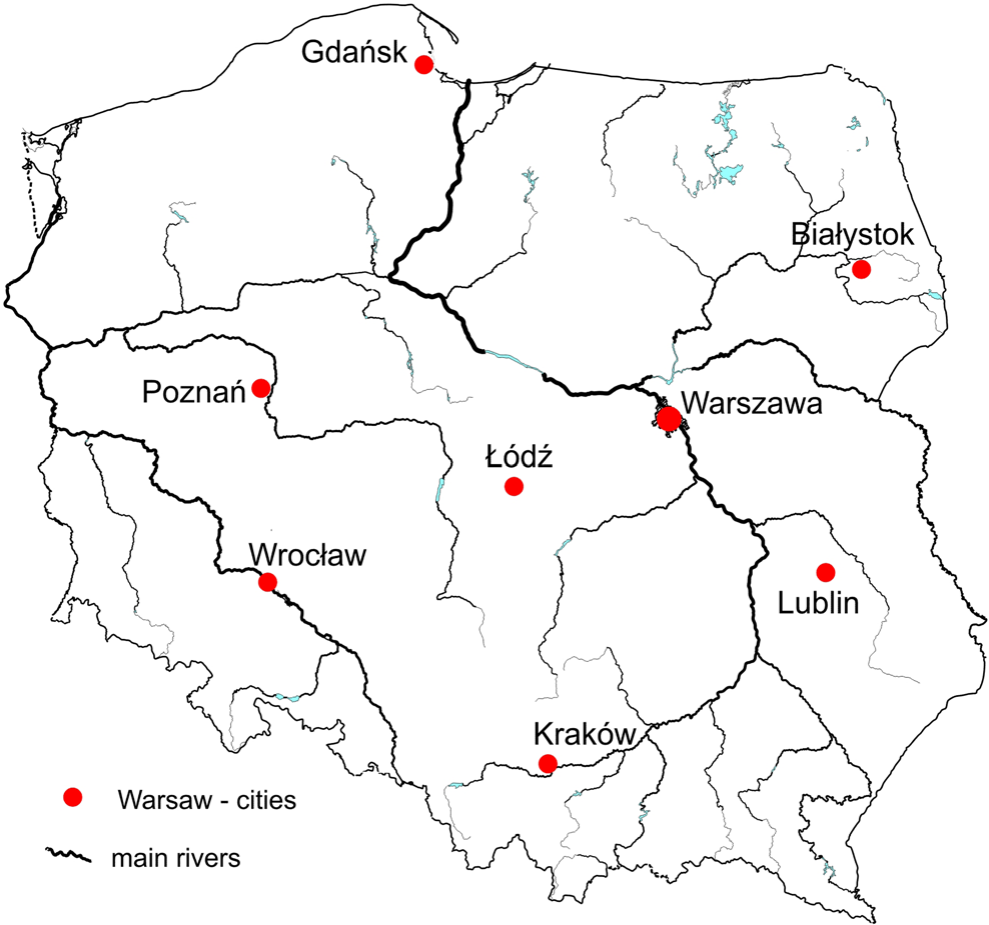
The analyzed cities

Daily mortality and meteorological data were analyzed for the years 1975–2014 in 8 Polish cities: Białystok, Gdańsk, Kraków, Lublin, Łódź, Poznań, Warszawa, and Wrocław. These are the largest Polish cities, with populations over 200,000 inhabitants and thus sufficient for statistical analysis of the number of deaths. The cities also represent different climatic regions of Poland (Fig. 1).

The meteorological data comprised daily mean, minimum and maximum air temperatures, and data at 12 UTC (air temperature, air humidity, wind speed, air pressure, cloudiness). They were used to calculate the Universal Thermal Climate Index (UTCI) with the Bioklima 2.6 software package (Błażejczyk and Błażejczyk 2010).

UTCI is based on the analysis of human heat balance using D. Fiala’s multi-segmental, multi-layered heat exchange model in given weather conditions. The model itself consists of two subsystems of heat exchange regulation: passive and active (Błażejczyk et al. 2012; Bröde et al. 2012; Fiala et al. 2012). In this analysis, all stress categories occurring when UTCI < − 13.0 °C were grouped as cold stress, and categories occurring when UTCI > 32.0 °C, as heat stress conditions.

The daily death numbers for the years 1975–2014 used in this study were provided by the National Institute of Public Health-National Institute of Hygiene. Mortality data comprised daily numbers of deaths from all causes, as well as from circulatory (390–459 in ICD-9 and I00-I99 in ICD-10 in the International Statistical Classification of Diseases and Related Health Problems) and respiratory system diseases (460–519 in ICD-9 and J00-J99 in ICD-10). All deaths were divided by sex (male, female) and age (under and above 65 years of age, including 65). These causes of death and age intervals are the most frequently analyzed in world literature in the context of dependence on thermal conditions (e.g., Kalkstein, Greene 1997; Huynen et al. 2001; Kuchcik 2003; Keatinge 2002; Kozłowska-Szczęsna et al. 2004; Rabczenko et al. 2009; Isaksen et al. 2016; Royé 2017). In recent years, as average life expectancy in Europe has been increasing, many studies have assumed higher age thresholds, e.g., 70 (Rabczenko et al. 2009) or 75 (Ruuhela et al. 2017). In Poland, 65 years is the retirement age for men and thus acts as a boundary (low pension, worse availability of private medical care). Previous studies have convinced the author that an age limit of 65 designating the elderly is reasonable in Polish conditions (Kuchcik 2001, 2003, 2006, 2017).

There is no reliable data about causes of deaths in the years 1997–1998 due to a strike of physicians in Poland.

The work did not analyze periods of influenza. Even though influenza-like illnesses could amount from 200,000 cases like in 2002 to 3,500,000 cases like in 2015 in Poland, the number of deaths for which flu was given as the main cause of death is only a dozen and they are included in respiratory deaths. It seems to be clearly underestimated and this is why the discussion on mortality in the winter season is very cautious. Furthermore, it should be mentioned that in the 65+ group, the incidence of influenza is the lowest and the highest is among children below 4 years of age (Wojtyniak et al. 2012b; Wojtyniak and Goryński 2016).

In the statistical analysis of strong thermal stress categories over the 40-year period, the TBATS 2 method was used to evaluate a time series data of complex seasonality (multiseasonality) (Gasparrini et al. 2012; QUANTUP 2014). Hockey-stick segmented regression was then used to assess the statistical significance of the data extracted by TBATS 2 and of the changes in thermally characteristic days. It estimated nonlinear parameters with two linear functions and two trends (1 and 2)—for the years before and after the year in which a trend changes direction. If finding a change point was not possible for a city, then a straight line was fitted to the data (only one trend). The significance of each slope was assessed. TBATS calculations were made using the R 3.3.1 “Forecast” package (Hyndman and Khandakar 2008):

1 $$ {Y}_t={B}_0+{B}_1\cdotp y+{B}_2\cdotp {\left(y- Ych\right)}^{+}\kern0.5em $$

2 $$ {\left(y- Ych\right)}^{+}=\left\{\begin{array}{c}0\kern4em for\ y\le Ych\\ {}y- Ych\kern0.75em for\ y> Ych\end{array}\right. $$

where:

*Y*_*t*_—number of characteristic days in a year,

*Ych*—year of the change,

*y*—given year,

*B*_*i*_—regression coefficients indicating the slope of the lines.

+—plus part of the equation.

In the hockey-stick method, only one point of change was set, despite awareness of the threat of overmatching the trend line to the data or assigning too much “weight” to individual extreme values.

Generalized additive models (GAM) were in turn used to investigate the relationship between UTCI and mortality. The multivariate model included the following possible confounding factors: general mortality trends, calendar year, day of the week, and daily mean air temperature. Taking long-term and seasonal changes into the model eliminates subsequent fears, e.g., increase in mortality at very low UTCI values is due to the highest mortality in winter months. The dependence of mortality on UTCI was modeled by cubic regression splines (Curriero et al. 2002; Wood 2006; Rabczenko et al. 2015, 2016) and had the form described by formula [3].

3 $$ \mathit{\ln}(Y)={\beta}_0+\sum \limits_{i=1}^ps\left({X}_i\right)+\sum \limits_{i=p+1}^k{\beta}_i{X}_i $$

where

*Y*—daily death number in the studied sub-population,

*X*_*i*_—*i*th factor in the model,

*s(.)*—factor whose impact on mortality is being modeled using the spline function,

*β*_*i*_—regression coefficient for factor being modeled using the linear function,

*k*—total number of factors in the model,

*p*—number of factors modeled using non-parametric functions.

First, the number of spline nodes to describe long-term and medium-term variations was chosen. Next, the dependence of mortality on UTCI value was determined and the results were presented on graphs. The relative risk of death (RR) was calculated with a 95% confidence interval.

RR is often used in epidemiological studies and illustrates a change of risk of death under the population exposure to the factor changes. It indicates rates of death in a given weather situation, as compared with the number of days with reference atmospheric conditions in the given city, also presented as the increase or decrease in predicted numbers of deaths in per cent. An increased or decreased risk of death on specific days compared with non-specific days occurred if the value “1” was outside the range of the confidence interval.

## Results

**Fig. 2:**
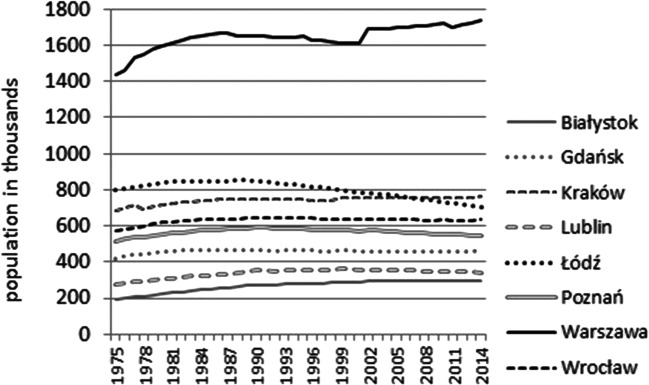
Population in the analyzed cities in the years 1975–2014

First, the populations of the 8 cities were considered. Over the 1975–2014 period, the biggest changes in population were noted in Wrocław and Gdańsk where in the 1970s only 6.5–7% of the population were aged over 65. These cities were newly resettled by mainly young persons displaced from the eastern territories ceded to the USSR after the Second World War and in the 1970, they were under 65. In Gdańsk, the number of deaths grew steadily from 650/100,000 citizens in 1975 to 1008/100,000 in 2013. Today, only the demographic situation in Łódź differs from that in the other cities. In Łódź, in 2014, persons over 65 years of age constituted 20% of the population and in this city, population aging is the highest among Polish cities with a population over 200,000 (Fig. 2).

In Poland, in the years 1975–2014, circulatory system deaths accounted for 46.4% of total deaths on average, while respiratory system deaths for 4.5%. However, while in the late 1980s and early 1990s, circulatory system deaths accounted for 50–55% of all deaths; in 2014, the respective figures were 40–45% depending on the city. Diseases of the respiratory system take fourth place among causes of death in Poland, after cancer and accidents and injuries. Their average share in total number of deaths varies from 3.6 in Kraków to 5.4% in Łódź. The highest overall mean rates of mortality per 100,000 people in the years 1975–2014 were those observed for Łódź (1321 deaths per 100,000) and Warszawa (1070). Meanwhile, the lowest value was 779 per 100,000 recorded in Białystok (Table 1).

**Table 1:** Yearly average number of deaths per 100 thousands citizens by cause of death and age (total, circ. from circulatory diseases, resp. respiratory diseases, *65+* total population and population over 65 years of age) and the mean percentage of circulatory and respiratory deaths in all cases, 1975–2014

City	Average number of deaths per 100,000 citizens						No. of given deaths/total (%)	
	total	total 65+	circ.	circ. 65+	resp.	resp. 65+	circ.	resp.
Białystok	779.0	509.4	349.7	281.8	33.7	25.3	44.6	4.3
Gdańsk	880.2	569.0	349.1	263.2	46.9	37.7	39.6	5.3
Kraków	921.9	640.9	451.9	372.2	32.8	25.5	49.0	3.6
Lublin	861.9	562.2	415.8	331.7	34.7	26.5	48.3	4.0
Łódź	1320.7	878.5	624.8	494.3	71.3	56.1	47.6	5.4
Poznań	1046.4	736.1	502.3	401.6	42.7	34.5	48.1	4.1
Warszawa	1070.2	747.5	484.2	396.1	55.0	45.0	45.2	5.2
Wrocław	903.3	581.0	443.9	341.8	36.8	26.3	49.2	4.1

The annual course of total mortality was similar in all cities. Winter (usually January) was characterized by a higher mortality rate and the summer season (always August) by a lower rate, the difference between them being 8–9%. The same course is typical for deaths from circulatory system diseases. Respiratory mortality was characterized by greater seasonal differences: the winter increase (January and February) reached 30–40% of the annual average and the summer decrease (August and September) was up to 25% of the annual average. However, the daily number of respiratory deaths was very small (Table 2).

**Table 2:** Daily average number of deaths from all causes, circulatory, and respiratory diseases in Warszawa, in the total population and among population over 65 years of age (65+), 1975–2014

Month	J	F	M	A	M	J	J	A	S	O	N	D	Year
All causes	3.18	3.13	3.08	2.91	2.85	2.78	2.78	2.69	2.79	2.92	2.94	3.12	2.93
All causes 65+	2.26	2.25	2.19	2.03	1.97	1.92	1.92	1.84	1.93	2.03	2.06	2.18	2.05
Circulatory	1.47	1.45	1.42	1.33	1.30	1.24	1.22	1.18	1.24	1.31	1.32	1.44	1.33
Circulatory 65+	1.21	1.20	1.17	1.09	1.06	1.00	1.00	0.96	1.01	1.07	1.09	1.16	1.08
Respiratory	0.20	0.20	0.19	0.16	0.13	0.12	0.12	0.12	0.12	0.14	0.15	0.17	0.15
Respiratory 65+	0.16	0.17	0.15	0.13	0.11	0.10	0.10	0.09	0.10	0.12	0.12	0.13	0.12

Big differences between numbers of circulatory and respiratory system diseases and overrepresentation of circulatory death are primarily due to the excessive use of “garbage codes” of deaths which are mainly from the group of cardiovascular diseases, like atherosclerosis. For example, the mortality rate due to atherosclerosis (I70) in Kraków and Wrocław was 6–9 times higher than in Białystok. That is why various coding for causes of deaths are most likely the reason for large regional differentiation of the causes of mortality in Poland (Wojtyniak et al. 2012a, b). This should be remembered in the analysis, but no changes can be made in the death cards and statistics. They are the only mortality statistics in Poland, even if they are imperfect. However, the present study does not analyze individual death causes, but the groups of them: circulatory and respiratory system diseases, and usually even the incorrectly given cause of death remains in the same group of diseases.

Mean yearly air temperature in the years 1975–2014 varied in Poland from 7.1 °C in Białystok (north-east) to 9.0 °C in Wrocław (south-west). Mean yearly UTCI calculated for 12 UTC varied from 2.1 °C in Gdańsk (coastal city) to 6.8 °C in Kraków (situated in the Vistula river valley in the south of Poland). Due to high wind speed on the coast, Gdańsk is characterized by the lowest number of days with UTCI > 32 °C and one of the highest numbers of days with UTCI < − 13 °C. By contrast, low wind speeds noted in the Vistula valley mean that heat stress is frequently observed in Kraków (Table 3).

**Table 3:** Thermal environment and trends (1 and 2—before and after the year of change of direction): analysis of days with heat stress (UTCI > 32 °C) and cold stress (UTCI < − 13 °C) in the analyzed cities in the years 1975–2014

City	*T*_mean_	UTCI yearly mean 12 UTC	UTCI threshold	Mean yearly number of days with UTCI	Trend characteristics		
					Year of change	Trend	Change days/year
Białystok	7.1	5.5	> 32 °C	4.8	1992	1	0.44*
						2	− 0.08
			< − 13 °C	44.9	1989	1	− 2.97*
						2	0.22
Gdańsk	8.2	2.1	> 32 °C	0.7	-	1	0.01
						2	–
			< − 13 °C	60.9	1989	1	− 3.31*
						2	0.42
Kraków	8.3	6.8	> 32 °C	8.4	1994	1	0.63*
						2	0.10
			< − 13 °C	42.8	-	1	0.11
						2	-
Lublin	7.6	4.7	> 32 °C	6.3	1993	1	0.40*
						2	− 0.02
			< − 13 °C	58.6	-	1	− 0.53*
						2	-
Łódź	8.3	4.4	> 32 °C	4.5	1994	1	0.39*
						2	− 0.13
			< − 13 °C	56.7	2000	1	− 1.42*
						2	1.39
Poznań	8.8	5.2	> 32 °C	5.6	1994	1	0.28
						2	0.03
			< − 13 °C	45.9	1992	1	− 1.67*
						2	0.56
Warszawa	8.4	4.3	> 32 °C	5.1	1993	1	0.33
						2	0.03
			< − 13 °C	61.2	-	1	− 0.62*
						2	-
Wrocław	9.0	6.7	> 32 °C	7.2	1983	1	0.43
						2	0.02
			< − 13 °C	36.8	1990	1	− 0.79
						2	0.00

*Statistical significance at *p* ≤ 0.05

**Fig. 3:**
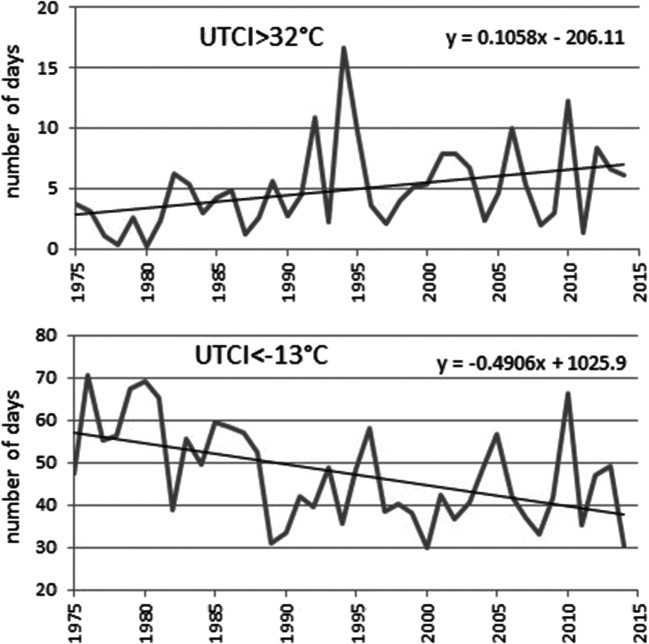
Average number of days with strong heat (UTCI > 32 °C) and cold (UTCI < − 13 °C) stress for the 8 cities in the years 1975–2014 with trend lines

The overall picture (Fig. 3) shows that in the years 1975–2014, the average number of days with UTCI > 32 °C for the 8 cities taken together changed from ca. 3 days in 1975 to 7 days in 2014 with two maxima in 1992 and 1994. The number of days with UTCI < − 13 °C has decreased from ca. 55 days in 1975 to 38 in 2014. However, cool years and cold winters are still being recorded, like in 2010 when 66 days with strong cold stress were recorded (Fig. 3).

Statistical analysis for individual cities using hockey-stick segmented regression has roughly confirmed the above picture. Most of the cities experienced a clear and often statistically significant increase in the frequency of thermal heat stress days (strong heat stress when UTCI is between 32.1 and 38.0 °C, and very strong heat stress with UTCI from 38.1 to 46.0 °C taken together) until 1992–1994: from + 0.3 to + 0.6 (Kraków) days per year. After this time, the frequency of heat stress days remained at a similar level (Table 3).

**Fig. 4:**
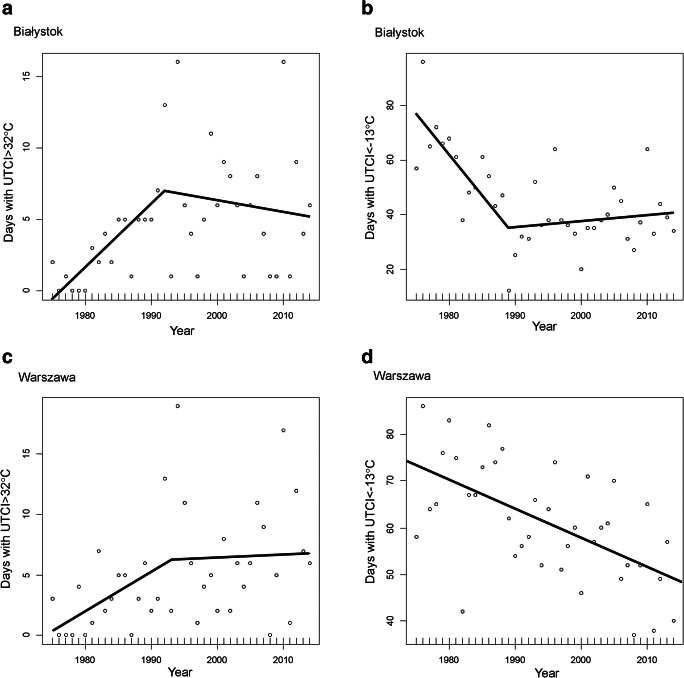
Trends for number of days with strong heat stress (UTCI > 32 °C) and strong cold stress (UTCI < − 13 °C) in the years 1975–2014 in Białystok and Warszawa

In turn, the number of days with UTCI < − 13 °C declined very markedly and was statistically significant until 1989 (in Białystok and Gdańsk) or even 2000 (in Łódź), with rates in the range of 0.8–3.3 days/year. However, since then, either the frequency of occurrence of such days increased slightly again by 0.2–1.4 days/year, or like in Lublin and Warszawa, the number of cold stress days declined steadily throughout the whole period, by 0.5 and 0.6 days/year respectively (Table 3, Fig. 4).

The relationship between mortality rates and UTCI in Poland is presented as regression curves show a shape half-way between a wide V and an inverted L. The parts of the curves on the side of low temperatures slope gently or sometimes do not rise at all, while those associated with high temperatures assume a steeper slope. This indicates a more rapid increase in mortality in a hot environment than in a cold one.

There is a clear climatic difference between cities located in warmer and colder parts of Poland. The “cool” cities (i.e., Gdańsk, Białystok, and Lublin located on the coast or in eastern Poland) were characterized by a clear thermal optimum. For UTCI between 5 and 30 °C, RR was lower than 1.0 which means that total mortality dropped up to 6% below the expected mortality. It turns out that the conditions under which mortality decreases include not only the “no thermal stress” range but also the “slight cold stress” and part of the “moderate heat stress” range (Fig. 4).

In “warm” cities (Łódź, Poznań, Warszawa, and Wrocław) “no thermal stress days” seemed to be completely neutral in context of mortality. The lowest mortality (RR under 1.0, less than expected) was noted under moderate or even strong cold stress conditions as measured by UTCI. The shape of the dependence of mortality on thermal environment calculated for Kraków places that city between “warm” and “cool” cities, but closer to “warm” cities.

**Fig. 5:**
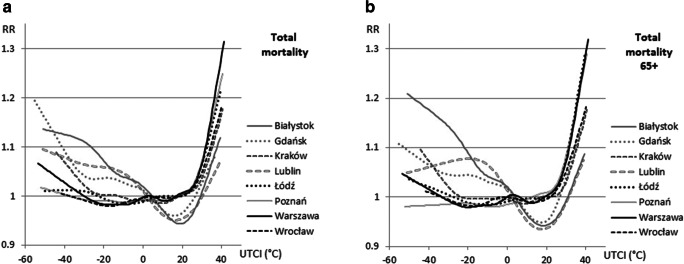
Relative risk of death (RR) due to all causes in the whole population (left) and among population over 65 years of age (65+) (right) and UTCI

In Wrocław, Poznań, and Łódź, no rise in mortality was noted under strong cold stress, while in Warszawa, Kraków, and Lublin, it did not exceed 10% under very strong und extreme cold stress. In turn, even moderate heat stress caused mortality to rise by 10% in central Poland (Warszawa, Poznań, Łódź). Under strong heat stress in most cities except Gdańsk and Lublin, the rise of RR was between 10 and 20%. Under very strong heat stress (UTCI > 38 °C), RR rose up to 25–30% in central Poland and only 15% in the warmest city, Wrocław. A UTCI of 41 °C entailed a 30% rise in total mortality in Warszawa. In “cool” cities, the rise of RR under heat stress was only 6–10% for UTCI above 37–38 °C (Fig. 5).

The impact of heat was slightly greater among people over 65 as compared with the whole population. In Łódź, Poznań, and Białystok, the RR under heat stress for this age group was 5–8% higher than among the total population. Furthermore, under strong heat stress, an increase in RR of 10%, 20%, or 30% respectively occurred for UTCI lower of 1 °C as compared with the whole population (Fig. 5).

**Fig. 6:**
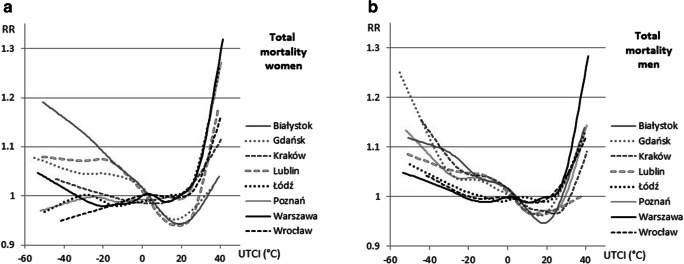
Relative risk of death (RR) due to all causes among women (left) and men (right) and UTCI

The difference between men and women was visible mainly in the response of the body to cold stress. In a cold environment, total mortality among women was at a markedly lower level than among men. In Poznań, Łódź, and Wrocław, RR was below 1 even under very strong cold stress. Only in the coldest Polish city (Białystok) did it rise to 19%, when UTCI < − 40 °C. In contrast to mortality under cold stress, mortality under heat stress grew strongly even by 25% in central Poland (Łódź, Warszawa, Poznań) at 38–39 °C UTCI. In Warszawa at 41 °C, UTCI mortality among women rose by 30% (Fig. 6).

As concerns mortality among men, a clear thermal optimum was noted in Gdańsk, Białystok, Lublin, Kraków, and Poznań (between 0 and 24 °C UTCI); however, in the other three cities, this was not observed. In days with strong cold thermal stress, RR of total mortality among men rose by 10% for UTCI below − 35 °C in four cities, while in the other four, the increase did not exceed 5–8%. Interestingly, the mortality curves for UTCI in Warszawa looked practically the same for men and women, but women’s RR in very strong heat stress was higher by c. 5% (Fig. 6).

**Fig. 7:**
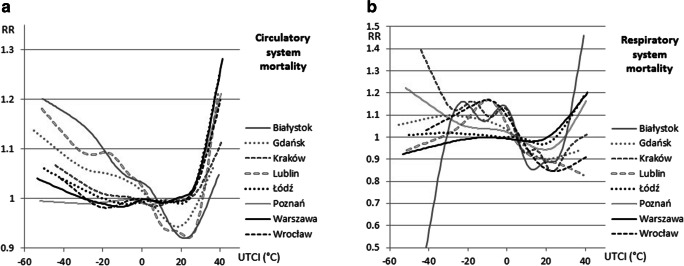
Relative risk of death (RR) due to circulatory system diseases in the whole population (left) and due to respiratory system diseases in the whole population (right) and UTCI

The regression for mortality due to diseases of the circulatory system against UTCI values looked very similar to total mortality. However, it was noted that the decline in circulatory mortality for UTCI in the 4–30 °C range in “cool” cities was 8% while the drop in total mortality was 6%, while the RR at low UTCI values in Białystok and Lublin (the coldest cities) was 6–9% higher than when considering all causes of mortality (Fig. 7).

**Fig. 8:**
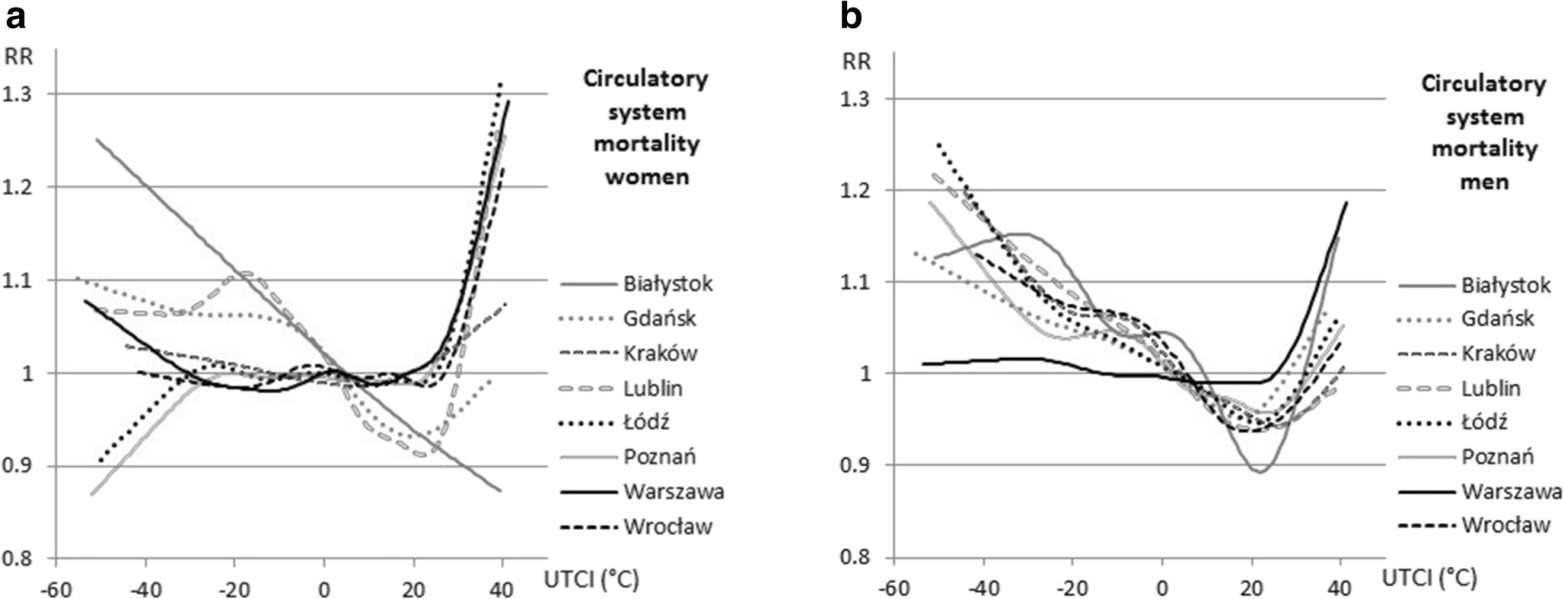
Relative risk of death (RR) due to circulatory system diseases among women (left) and men (right) and UTCI

Great differences were noted between men and women’s mortality from circulatory system diseases. In summer in central Poland, a 10% increase in morality among women appeared at 31 °C, though in Wrocław and Lublin, this occurred at a UTCI of 34 °C. Very strong heat stress involved a rise in RR of over 20% in Poznań and Wrocław and a 30% rise in Łódź. The regression curve in Białystok took a different shape with no increase in mortality on heat stress days. With respect to circulatory disease mortality among men, RR rose by 13–25% depending on the city under extreme cold stress but only by 2–6% under strong heat stress. Again, this relationship differed for Białystok and Warszawa. The male population in the latter reacted to cold stress more like women did—the increase in mortality was noted only in a hot environment, not in a cold one (Fig. 8).

The correlation of respiratory mortality and UTCI clearly differed from the others, probably because of the small number of cases analyzed in regression. The differences between cities were bigger, and the changes in RR higher. For example, in Białystok, extreme and very strong cold stress was noted on 5 days on average, another 5 days were characterized by strong and very strong heat stress. Therefore, with an average daily respiratory death number equal to 0.2, it is possible for no respiratory deaths to have occurred on such days or that there were suddenly such 2–3 deaths 1 day and RR jumped to a level of 1.5 (Fig. 7).

## Discussion

This study has shown a significant decrease in the frequency of cold stress days over the whole 1975–2014 period, and a high increase in strong heat stress days until 1994 and a steady rise or stabilization afterwards. The trends for the number of extreme heat/cold stress days in Poland in the period 1975–2014 have confirmed the results of Błażejczyk et al. (2015). In this study, at the beginning of the analyzed period, cold stress days with UTCI < − 13 °C were 18 times more frequent than heat stress days with UTCI > 32 °C, though in 2014, the same ratio was only 5. Cold stress days observed decreased from ca. 55 in 1975 to 38 in 2014, and the number of heat stress days grew from 3 to 7 in the same years. In turn, over the years 1973–2012 (UTCI average for Poland), these ratios were respectively 12 and 3: the occurrence of cold stress days decreased from ca. 58 to 32, and the number of heat stress days increased from 2 to 10 (Błażejczyk et al. 2015).

There is no other such long-term studies investigating UTCI and mortality to which the results of these studies could be compared, and the only possibility is to compare them with air temperature—mortality studies which are usually carried out as part of large projects by big, international teams (Baccini et al. 2008; Guo et al. 2014; Gasparrini et al. 2015). The shape of the regression lines for UTCI-related mortality is between a U and a V or an inverted L, with a long, but gentle slope on the side of low temperatures and a steep, mostly shorter part on the side of high temperatures. These relationships are similar to those found in the PHEWE project (assessment and prevention of acute health effects and weather conditions in Europe) for Helsinki, Stockholm, Praha, and Zurich. In this project, mortality was related not to air temperature but to apparent temperature (Steadman 1984; Kalkstein and Valimont 1986), which is another biometeorological index used quite often in weather-mortality studies (Analitis et al. 2008; Baccini et al. 2008).

This UTCI-mortality study showed clear differences between 3 cities located in the eastern, cold part of Poland (Białystok, Lublin) and on the coast (Gdańsk) called “cool”, and 4 cities called “warm” located in central and western Poland (Łódź, Poznań, Warszawa, and Wrocław). “Cool” cities were characterized by a clear thermal optimum accompanying UTCI in the 5–30 °C range, while in “warm” cities, there was no clear optimum. This corresponds to research from the USA for the years 1973–1994 and also based on daily data and using the GAM smooth function and a cubic spline function. In the US research, northern (colder) cities were characterized by a clear thermal optimum with significantly minimum mortality rate while in southern (warmer) cities, no such feature was observed (Curriero et al. 2002).

Interestingly, in the “warm” cities situated in central Poland, the lowest daily mortality rates occurred on days with moderate or even strong cold stress. This suggests that UTCI stress categories do not exactly match the Polish climate and when considering mortality, it is more reasonable to use the UTCI value itself than the selected stress categories.

The difference between the male and female organism’s response to thermal environment was seen in the much higher increase in mortality among men under strong cold stress than under heat stress, in all cities except Warszawa. When UTCI was below − 20 °C, men’s mortality, especially from circulatory system diseases, was ca. 10% higher than women’s mortality. By contrast, women’s mortality rose fast under strong heat stress. One of the reasons for that could be different reactions to heat and cold of men and women. Men and more athletic people have significantly more efficient heat exchange with the environment in the process of sweating: they sweat more heavily and at a lower temperature. Women’s sweat glands are activated at higher skin temperatures than men’s. This is why women could be more susceptible to overheating in severe heat stress (Hajat et al. 2007; Rey et al. 2009; Ichinose-Kuwahara et al. 2010; Cheshire 2016), that is, particularly visible in RR of circulatory system diseases higher among women of 7% in Warszawa, 12% in Wrocław, and 15% in Poznań comparing with men. In a cold environment, a woman’s body keeps proper internal temperature longer than a man’s and this applies also to older women, mainly due to a higher proportion of fat in women and a smaller body surface area where heat is exchanged (Wagner and Horvath 1985; Young et al. 1996). Among all deaths, there are also those due to hypothermia of intoxicated or/and homeless people, of which over 83% are men (Homeless 2019). There are many other factors, e.g., men are more likely to work outdoors, even during winter.

In Warszawa, men’s mortality was similar to women’s mortality. The excess of male deaths under heat stress was the same as among women (20%) and it did not occur under cold stress, which is very interesting. There is no easy explanation for this. Some of the reasons could be the different lifestyle of men in the capital city, working late in offices, highest GDP in Poland, and significantly lower alcohol consumption compared with the rest of the country (TNS 2013; GUS 2014).

Thermoregulation processes become less effective with age and therefore older people are more sensitive to extreme heat and cold (Kenney and Munce 2003). Five to 8% higher mortality rates among people over 65 years of age compared with the whole population were noted. In Helsinki ,in the period 1972–2014 (i.e., almost the same as studied here), the highest increase in mortality under extreme thermal conditions (determined by the PET index) was found in the group over 75 years of age (Ruuhela et al. 2017) and this increase was almost the same as the one shown in the present paper: between 5 and 8% compared with the total mortality. Other polish studies of thermal conditions and mortality also indicate that elderly people are a group that is more vulnerable to extreme cold and heat (Kozłowska-Szczęsna et al. 2004; Rabczenko et al. 2009).

In the future, it would be interesting to include information about the socio-economic status of deceased/sick people; however, no such data is being commonly collected in Poland. Such information could clarify the impact of the environment itself on the human body. In the next studies concerning the relationship between weather parameters and mortality, the periods of increased influenza cases should be taken into models, even if they are not directly reported in mortality statistics.
